# NKG2C^+^NKG2A^−^ Natural Killer Cells are Associated with a Lower Viral Set Point and may Predict Disease Progression in Individuals with Primary HIV Infection

**DOI:** 10.3389/fimmu.2017.01176

**Published:** 2017-09-20

**Authors:** Meichen Ma, Zhuo Wang, Xi Chen, Anfu Tao, Lei He, Shuai Fu, Zining Zhang, Yajing Fu, Chenxi Guo, Jing Liu, Xiaoxu Han, Junjie Xu, Zhenxing Chu, Haibo Ding, Hong Shang, Yongjun Jiang

**Affiliations:** ^1^Key Laboratory of AIDS Immunology of National Health and Family Planning Commission, Department of Laboratory Medicine, The First Affiliated Hospital, China Medical University, Shenyang, China; ^2^Collaborative Innovation Center for Diagnosis and Treatment of Infectious Diseases, Hangzhou, China

**Keywords:** NKG2C, NKG2A, pHi, HIV, prediction

## Abstract

Natural killer (NK) cells are the first line of defense against pathogens of the immune system and also play an important role in resistance against HIV. The activating receptor NKG2C and the inhibitory receptor NKG2A co-modulate the function of NK cells by recognizing the same ligand, HLA-E. However, the role of NKG2A and NKG2C on viral set point and the prediction of HIV disease progression have been rarely reported. In this study, we determined the expression of NKG2C or NKG2A on the surface of NK cells from 22 individuals with primary HIV infection (PHI) stage and 23 HIV-negative normal control (NC) subjects. The CD4^+^ T cell count and plasma level of HIV RNA in the infected individuals were longitudinally followed-up for about 720 days. The proportion of NKG2C^+^NKG2A^−^ NK cells was higher in subjects from the low set point group and was negatively correlated with the viral load. In addition, strong anti-HIV activities were observed in NKG2C^+^ NK cells from the HIV-positive donors. Furthermore, a proportion of NKG2C^+^NKG2A^−^ NK cells >35.45%, and a ratio of NKG2C/NKG2A >1.7 were predictive for higher CD4^+^ T cell counts 720 days after infection. Collectively, the experimental results allow us to draw the conclusion that NKG2C^+^ NK cells might exert an antiviral effect and that the proportion of NKG2C^+^NKG2A^−^ NK cells, and the ratio of NKG2C/NKG2A, are potential biomarkers for predicting HIV disease progression.

## Introduction

Natural killer (NK) cells are very important in immune surveillance and contribute to host resistance against viruses and tumors (1–3). NK cells may also serve to control HIV infection in long-term non-progressors (LTNPs), as well as individuals who have been to exposed to HIV but remain uninfected (4–7).

Natural killer cells express a large number of inhibitory or activating receptors, such as NKG2 receptors and killer-cell immunoglobulin-like receptors, and bind to their cognate ligands on the surface of target cells (8). NKG2C or NKG2A forms a heterodimer with C-type lectin CD94, and recognizes the non-classical major histocompatibility complex class I molecule, HLA-E (9). NKG2C delivers activating signals through immunoreceptor tyrosine-based activation motifs at the cytoplasmic tails of DAP12 transmembrane proteins (10), while NKG2A appears to inhibit NK activation *via* immunoreceptor tyrosine-based inhibitory motifs in the cytoplasm (11). Previous studies have shown that the inhibitory receptor NKG2A has a higher affinity for ligands, which is possibly correlated with the presumed dominance of inhibitory signals over activating signals (9, 12, 13).

Alterations in the expression of NKG2C or NKG2A have been observed in many diseases. In the mouse model, Ly49H (equivalent to NKG2C in humans) can directly recognize the mouse cytomegalovirus antigen and play a role in defending against infection (14, 15). In humans, NKG2C has been described as human cytomegalovirus (HCMV) specific, and positive HCMV serology has been associated with the proportion of NKG2C^+^ NK cells (16–18). In addition, NKG2A has been reported to be upregulated in chronic hepatitis B infection (19).

The expression of NKG2C and/or NKG2A on NK cells has been reported in chronic or early HIV-infected patients. The expression of NKG2C in HIV-infected LTNPs was found to be higher than that of progressors, although there was no significant difference in the expression of NKG2A (20). However, Ballan et al. found no difference for the proportion of NKG2C expression when compared between HIV^+^ and HIV^−^children (21). Furthermore, Lima et al. reported that HIV-exposed seronegative individuals, HIV-infected individuals, and healthy control subjects all showed a high correlation between the concentration of anti-Cytomegalo virus (CMV) IgG antibody and the proportion of CD56^dim^NKG2C^+^ cells (22). Guma et al. also observed that the elevated proportions of NKG2C^+^ NK cells in HIV-1-positive patients were related to a concomitant HCMV infection (23).

However, studies relating to NKG2C and NKG2A expression in primary HIV-infected patients are rarely reported. The primary stage of HIV infection is very important, because the viral set point is formed during this stage, which determines the subsequent progression of HIV disease. The expression of NKG2C or NKG2A on the surface of NK cells from individuals with primary HIV infection (PHI), and their predictive roles for HIV disease progression, is needed to be elucidated.

In this study, we investigated NKG2C and NKG2A expression on the surface of NK cells and evaluated the role of such expression in the suppression of HIV replication. We also performed a survival analysis to explore the relationship between NKG2C or NKG2A expression and HIV disease progression. We found that in primary HIV-infected patients, the proportion of NKG2C^+^ NK cells was increased compared with normal controls (NCs) and that the proportion of NKG2C^+^NKG2A^−^ NK cells was correlated with HIV viral set point. We also proved that after stimulation, NKG2C^+^ NK cells had a stronger ability to secrete IFN-γ and express CD107a than NKG2C^−^ NK cells. Moreover, we found that the proportion of NKG2C^+^NKG2A^−^ NK cells, and the ratio of NKG2C/NKG2A, may represent useful biomarkers to predict the progression of HIV disease.

## Materials and Methods

### Study Population

This study enrolled a total of 45 individuals, 22 of whom had a PHI and were being seen at the Red Ribbon Clinic in the First Affiliated Hospital of China Medical University. The HIV-infected individuals were all males, aged 19–52 years; 23 HIV-negative individuals served as NC subjects. The inclusion criteria for PHI subjects were as follows: aged >18 years; infected with HIV within 36–156 days at enrollment; antiretroviral therapy (ART) naïve; and followed-up for 720 days. The inclusion criteria for NC subjects were as follows: HIV-negative; normal blood routine examination and normal liver function; negative for hepatitis B and hepatitis C antibody; and no disease of the immune system (Table 1). The subjects had been tested for anti-CMV IgG antibody, anti-CMV IgM antibody, and CMV nucleic acid. The demographic, immunological, virological, and CMV infection data of all study subjects are shown in Table 1. The Research and Ethics Committee of The First Affiliated Hospital of China Medical University approved the protocol for this study, and each enrolled individual provided their written informed consent for participation in the study.

**Table 1:** Characteristics of study participants.

	Normal control (NC), *N* = 23	Primary HIV infection (PHI), *N* = 22	PHI followed-up for 720 days, *N* = 19	*p*^b^
Age^a^ (years)	39 (33–46)	27 (19–52)	30 (21–54)	>0.05
CD4^+^ T cells^a^ (μL)	899 (454–1,460)	550 (202–877)	378 (147–813)	<0.0001
Sex	Male	Male	Male	
Plasma level of HIV RNA^a^ (copies/mL)	–	20,200 (20–472,000)	12,500 (20–200,248)	
Infection time^a^ (day)	–	94 (36–156)	733 (680–806)	
Cytomegalo virus (CMV)-IgG status	90.9% (10/11)	100% (21/21)		>0.05
CMV-IgM status	9.09% (1/11)	9.52% (2/21)		>0.05
CMV-DNA status	0 (0/11)	0 (0/21)		

*All PHI subjects were ART naïve*.

*^a^Data were showed in median with range*.

*^b^The comparison of NC and PHI*.

### Antibodies

We used the following antibodies in this study: NKG2C-APC, NKG2C-PerCp, NKG2A-PE (R&D); CD3-FITC, CD4-APC-Cy7, CD16-PerCP-Cy5.5, CD56-PE-Cy7, CD107a-APC-H7 (BD Biosciences, San Jose, CA, USA); IFN-γ-BV421 (Biolegend, San Diego, CA, USA); CD4-FITC/CD8-PE/CD3-PerCP (BD Biosciences, San Jose, CA, USA).

### Determination of NKG2C and NKG2A Expression

**Figure 1:**
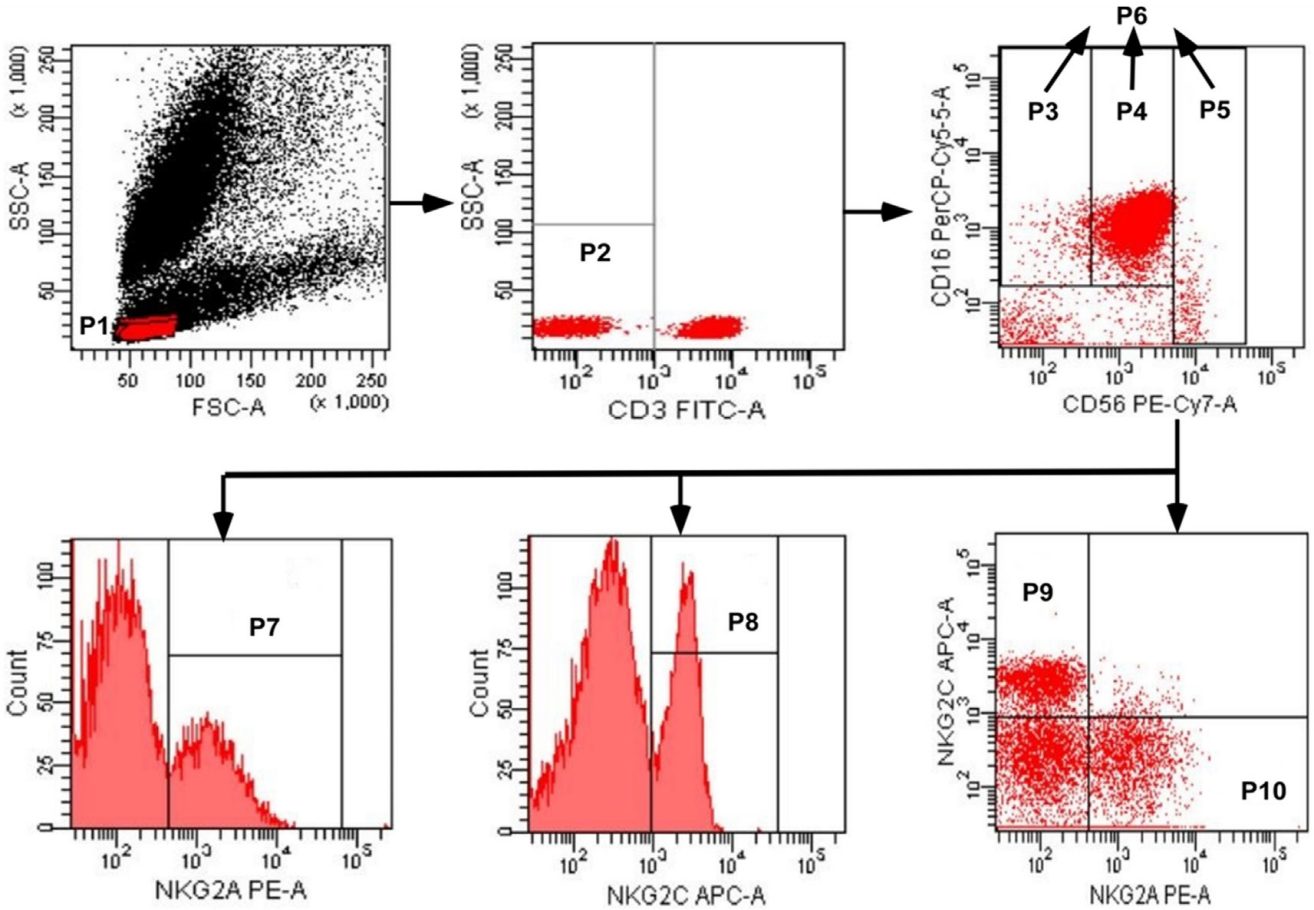
Gating strategy used to identify natural killer (NK) cell populations. The lymphocytes were gated using aside and forward scatter dot plot (P1). NK cells were identified from the CD3^−^ gate (P2) on the basis of CD16 and CD56 expression. The three NK cell subsets were gated as CD56^neg^CD16^+^ (P3), CD56^dim^CD16^+^ (P4), and CD56^bright^CD16^+/−^ (P5). The total population of NK cells (P6) was composed by the three subsets (P3 + P4 + P5). The total population of NK cells, and the three subsets, were analyzed for the surface expression of NKG2C and NKG2A. NKG2A^+^ NK cells, NKG2C^+^ NK cells, NKG2C^+^NKG2A^−^ NK cells, and NKG2A^+^NKG2C^−^ NK cells were identified in the P7, P8, P9, and P10 regions, respectively.

A whole blood sample was collected from each subject by venipuncture and a cocktail of fluorescent antibodies was used against CD3, CD16, CD56, NKG2C, and NKG2A. Mixtures were incubated for 30 min at 4°C, and erythrocytes were lysed using red blood cell lysing solution. Three NK cell subsets were identified as CD3^−^CD56^−^CD16^+^, CD3^−^CD56^dim^CD16^+^, and CD3^−^CD56^bri^CD16^+/−^, and the total population of NK cells included these three subsets (Figure 1). The expression of NKG2A or NKG2C was detected on NK cell subsets, and total NK cells were analyzed by flow cytometry; digital data from scatter plots were analyzed with Diva software.

### Detection for IFN-γ Secretion and CD107a Expression

Freshly isolated peripheral blood mononuclear cells (PBMCs) were incubated in the presence of phorbol 12-myristate 13-acetate (0.1 µg/mL) and ionomycin (5 µg/mL) at 37°C for 6 h. The cells were then stained for a panel of cell-surface markers (CD3-PerCP, CD56-PE-Cy7, NKG2C-APC, and CD107a-APC-H7). Intracellular staining was performed by incubating cells in 1× Perm/Wash Buffer (Becton Dickinson) for 15 min in the dark, and then with BV421-conjugated anti-IFN-γ (Biolegend, USA) for 30 min at 4°C. After staining, the cells were fixed in 1% formaldehyde; CD107a and IFN-γ expression were detected by flow cytometry.

### Detection of the Anti-HIV Activity of NKG2C^+^ NK Cells

CD4^+^ T cells, NKG2C^+^ NK cells, and NKG2C^−^ NK cells were sorted from PBMCs of HIV-negative donors (*N* = 5) and HIV-positive donors (*N* = 7) by BD FACS Aria. CD4^+^ T cells were stimulated by PHA (50 ng/mL, Sigma) and IL-2 (20 U/mL, Sigma) for 36 h, while NK cells were stimulated by IL-12 (10 ng/mL, R&D), IL-15 (50 ng/mL, R&D), and IL-18 (100 ng/mL, R&D) for 36 h. CD4^+^ T cells were infected by HIV-1 NL4-3 strain and co-cultivated with autologous NKG2C^+^ NK cells, or NKG2C^−^ NK cells, at a target ratio (E:T) of 5:1 for 5 days. Supernatants were collected from each culture and tested for HIV-1 P24 antigen by ELISA (QuantoBio, Beijing, China).

### Detection of CD4^+^ T Cell Counts

CD4^+^ T cell counts were determined with a FACS Calibur flow cytometer (Becton Dickinson). A single-platform lyse-no-wash procedure was conducted with TriTEST anti-CD4-FITC/CD8-PE/CD3-PerCP reagents and Trucount tubes (Becton Dickinson).

### Measurements of Plasma HIV RNA Levels

Plasma HIV RNA levels were determined by reverse transcription polymerase chain reaction (RT-PCR) performed with the COBAS^®^AmpliPrep^®^/COBAS Taqman system (Roche Diagnostic Systems); this assay had a detection range of 20–10,000,000 copies/mL. HIV RNA copy numbers were calculated by using the manufacturer’s reference standards.

### CMV Detection

Levels of IgM and IgG against HCMV in plasma samples were detected with a chemiluminescence immunoassay (LIAISON^®^CMV IgM II, LIAISON^®^ CMV IgG II, DiaSorin S.p.A., Saluggia, Italy). HCMV DNA was analyzed with an RT-PCR Kit (CMV Real Time PCR Kit, Liferiver, Shanghai, China). All samples were tested in the Department of Clinical Laboratory, at the First Affiliated Hospital of China Medical University.

### Statistical Analysis

Statistical comparisons of quantitative data between two groups were performed using the Mann–Whitney *U* test. The Kruskal–Wallis test was additionally used to compare significant differences across three groups. Associations between two groups were determined by the Spearman’s rank test. The non-parametric Wilcoxon matched-pairs signed-rank test was used to compare between two groups. Chi-square tests were conducted to examine differences in qualitative analysis. Receiver operating characteristic (ROC) curves and Kaplan–Meier survival analysis were used to evaluate the influence of NKG2C and NKG2A expression on NK cells upon the decline of CD4^+^ T cell counts. A *p*-value < 0.05 (two-tailed test) was considered to be statistically significant. All data analysis was performed using SPSS 20.0 (for three groups) and GraphPad Prism Version 5.0 software (for rest of data).

## Results

### The Proportion of NKG2C^+^ NK Cells was Higher in PHI Individuals

**Figure 2:**
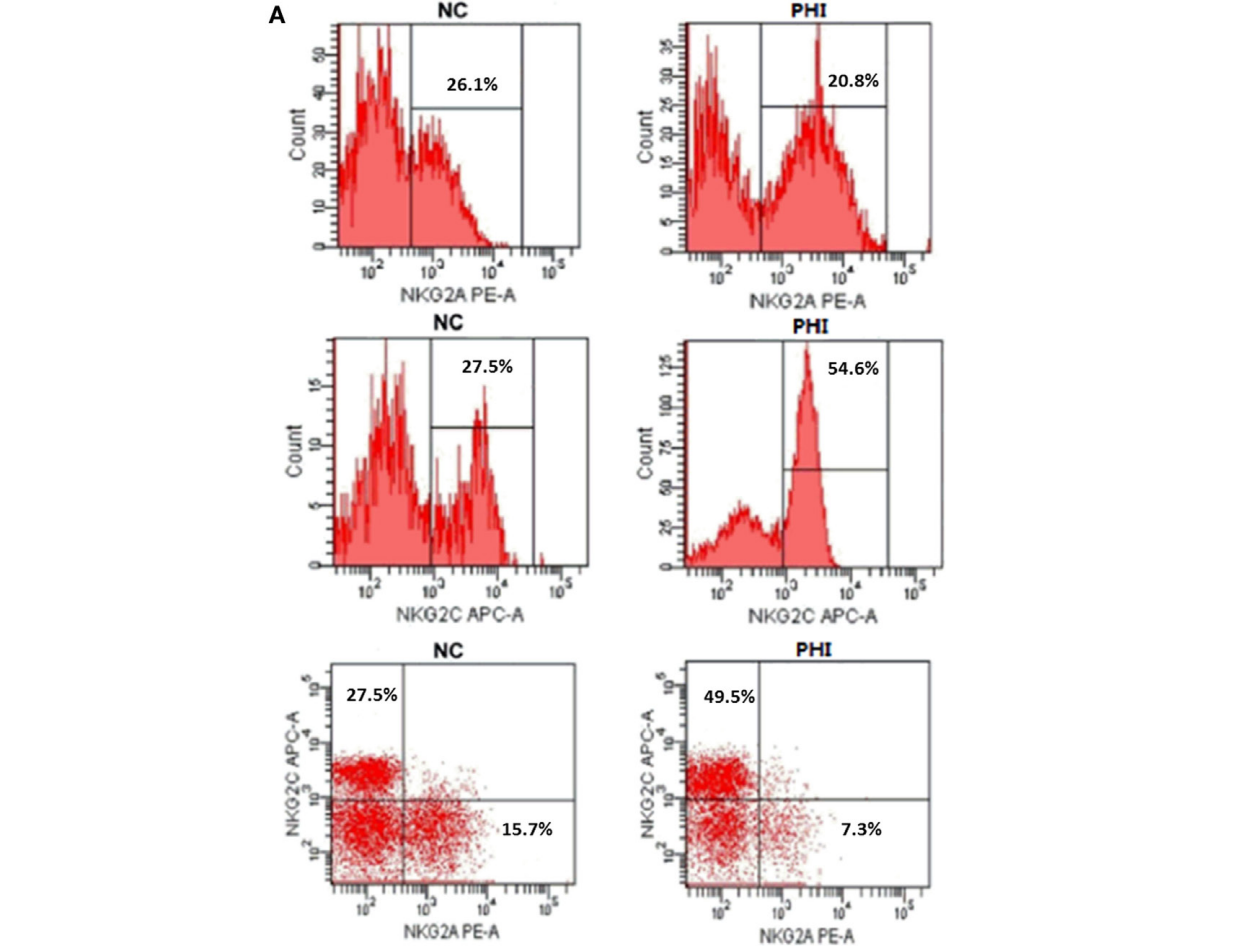

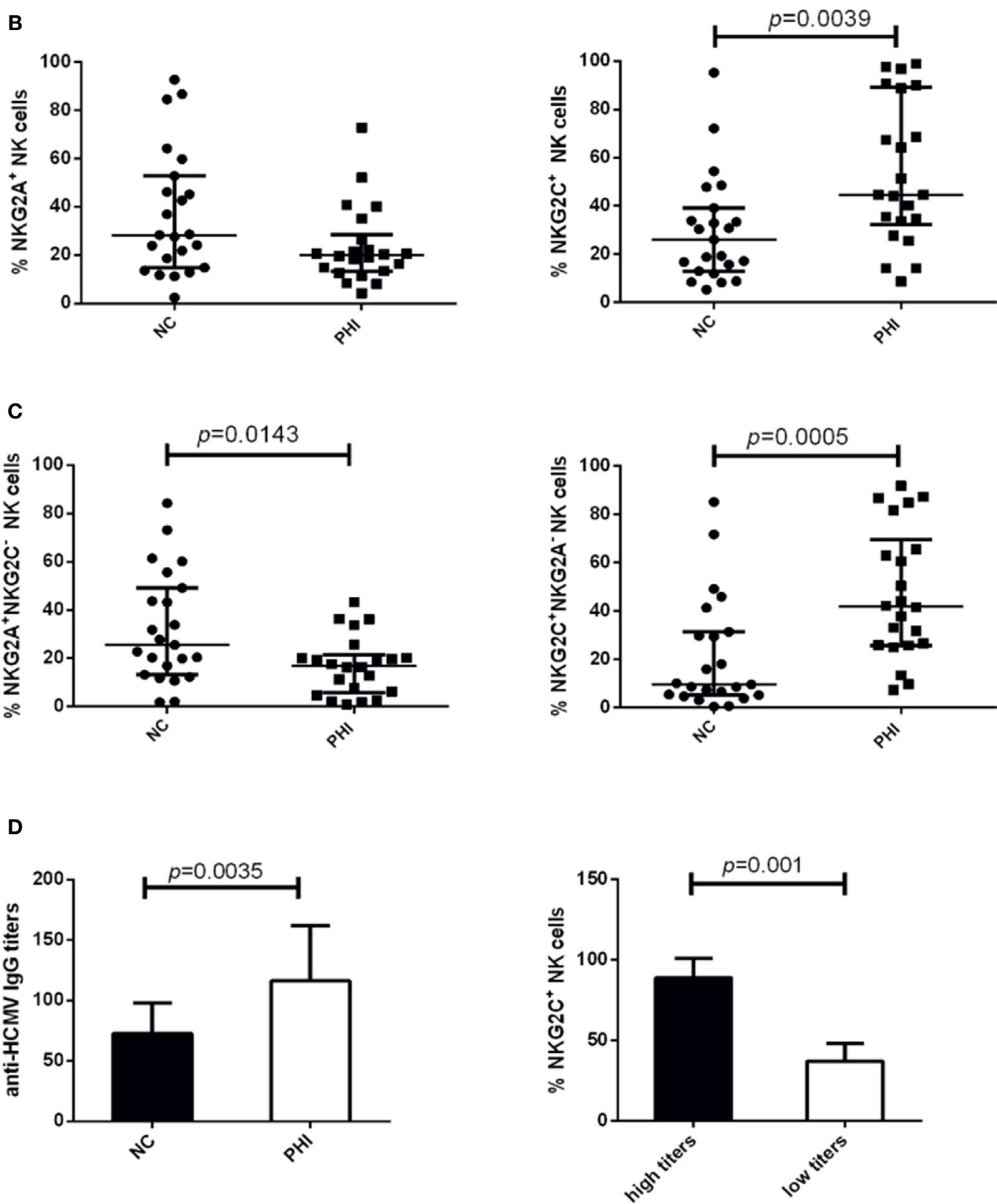
Alterations of NKG2C and NKG2A expression on natural killer (NK) cells in primary HIV infection (PHI) and normal control (NC) subjects. **(A)** Representative flow cytometry plots of single expression and co-expression of NKG2A and NKG2C on NK cells from NC and PHI subjects. **(B)** Comparison of the proportions of NKG2A^+^ or NKG2C^+^ NK cells between NC and PHI subjects. **(C)** Comparison of the proportions of NKG2A^+^NKG2C^−^ and NKG2C^+^NKG2A^−^ NK cells between NC and PHI subjects. **(D)** Comparison of the levels of anti-human cytomegalovirus (HCMV) IgG titers between NC and PHI subjects. The proportions of NKG2C^+^ NK cells were compared between high and low anti-HCMV IgG titer groups. Mann–Whitney tests were used to compare groups, and *p* values are shown where *p* < 0.05. Error bars indicate median and interquartile range.

To investigate the alteration of NKG2A and NKG2C expression on NK cells during HIV infection, we conducted the assessment with flow cytometry, and the representative flow cytometry plots were showed in Figure 2A. First, we detected the single expression of NKG2A and NKG2C on NK cells of PBMCs from PHI and age-matched NC subjects. Results showed that the proportions of NKG2A expression were comparable between NC and PHI subjects (*p* > 0.05; Figure 2B). However, the proportions of NKG2C expression were higher in PHI subjects than in the NC subjects (*p* = 0.0039; Figure 2B). Most of the literature only tested the single expression of NKG2A or NKG2C on NK cells, but not the co-expression of these receptors. NKG2A^+^ NK cells can be either NKG2A^+^NKG2C^−^ NK or NKG2A^+^NKG2C^+^ NK cells. However, NKG2A and NKG2C play opposing roles in terms of NK cell function, and the function of NKG2A^+^ NK cells is likely to differ from that of NKG2A^+^NKG2C^−^ NK cells. Therefore, in this study, we deliberately evaluated the co-expression of NKG2A and NKG2C on NK cells. We found that the proportion of NKG2A^+^NKG2C^−^ NK cells was significantly lower in PHI subjects than that in NC subjects (*p* = 0.0143; Figure 2C), although there was no significant difference in terms of the expression of NKG2A alone. In addition, we also found that the proportion of NKG2C^+^NKG2A^−^ NK cells was significantly higher in PHI subjects than that in NC subjects (*p* = 0.0005; Figure 2C). Similar results were observed for three NK cell: CD56^dim^CD16^+^, CD56^bri^CD16^+/−^, and CD56^−^CD16^+^ (Figures S1A–C in Supplementary Material). We also tested the mean fluorescence intensity of NKG2C or NKG2A expression and found that there were no significant differences when compared between PHI and NC subjects (data not shown). As most people are infected by HCMV in a latent manner, we investigated the association between the proportion of NKG2C^+^ NK cells and HCMV infection. We tested anti-HCMV IgG, IgM, and nucleic acid of HCMV; and the data showed that there was no significant difference on the positive rates between NC and PHI subjects (Table 1). However, we found that the titers of anti-HCMV IgG were significantly higher in PHI subjects compared with NC subjects (*p* = 0.0035), and the proportions of NKG2C^+^ NK cells were upregulated in PHI subjects along with a higher titers of anti-HCMV IgG (*p* = 0.001; Figure 2D). We further removed the PHI subjects with current HCMV infection (positive for anti-HCMV IgM antibody) and compared the proportion of NKG2C^+^ NK cells between NC subjects and the remaining PHI subjects. This analysis confirmed that the proportions of NKG2C^+^ NK cells were significantly higher in PHI subjects without current HCMV infection (*p* = 0.0066; data not shown). Overall, these data showed that NKG2C^+^ NK cells expanded during PHI, and we further investigated their potential roles in HIV infection.

### The Proportion of NKG2C^+^NKG2A^−^ NK Cells was Higher in the Low Viral Set Point Group of PHI Subjects and was Negatively Correlated with Plasma Levels of HIV RNA

**Figure 3:**
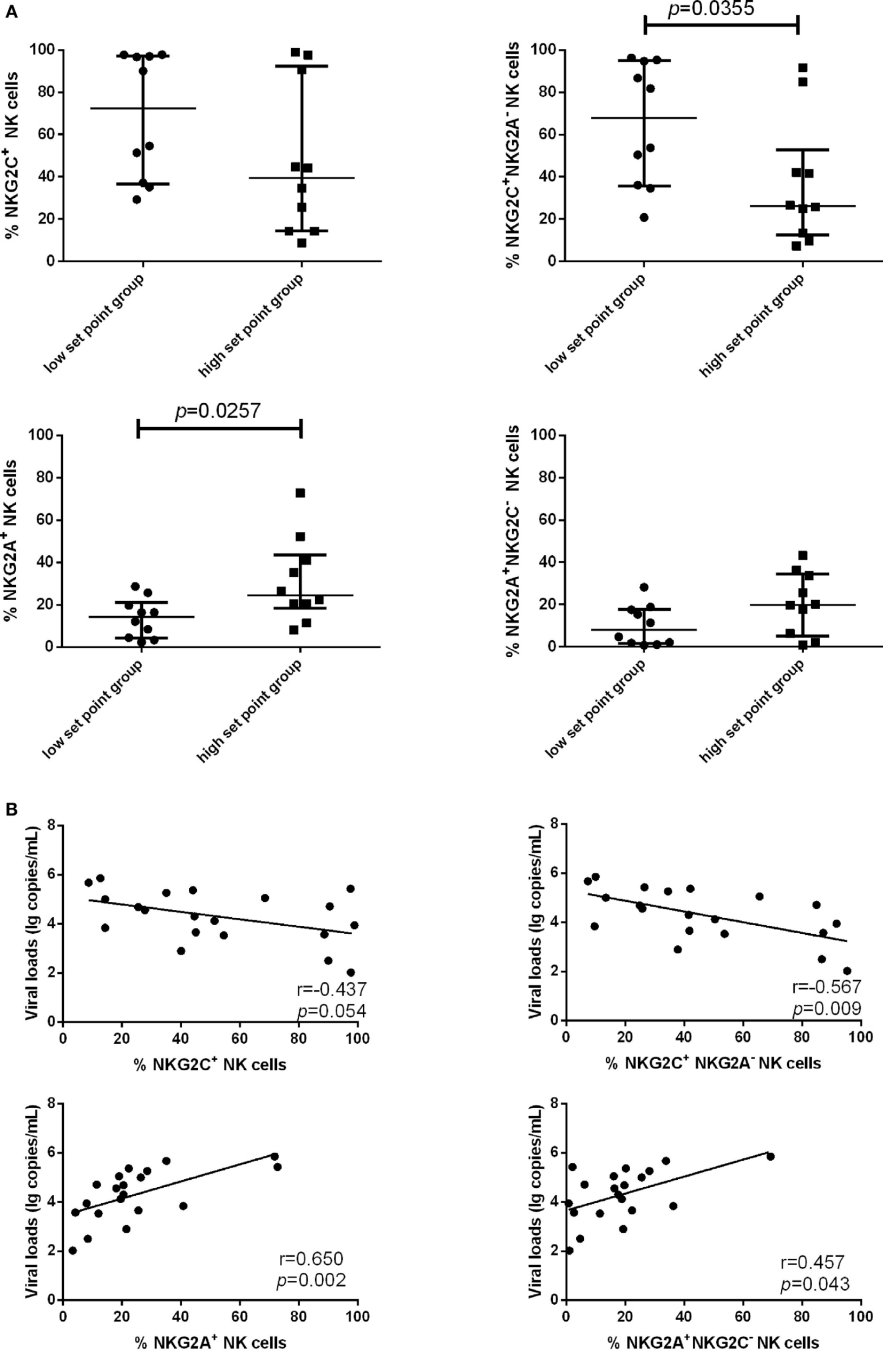

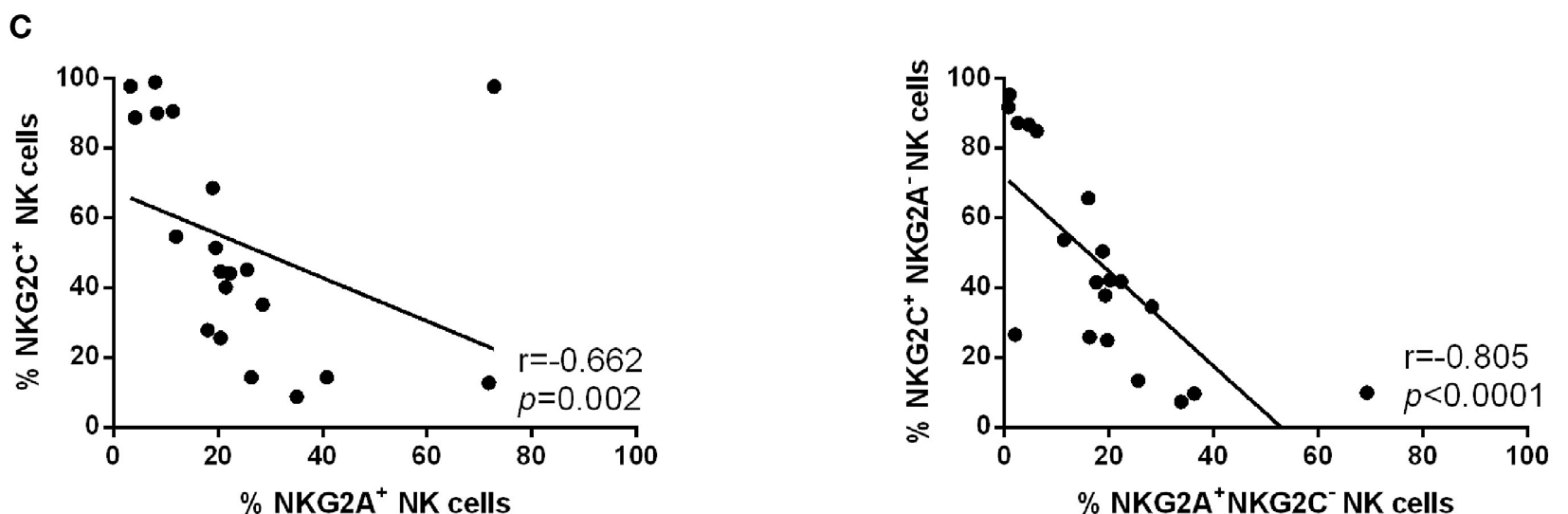
Associations between the expression of NKG2C or NKG2A on natural killer (NK) cells and viral set point or viral load. **(A)** Comparison of the relative proportions of NKG2C or NKG2A expression on NK cells between the low set point group (<10^4^ copies/mL) and the high set point group (>10^4^ copies/mL). **(B)** Correlation analysis between NKG2C or NKG2A expression on NK cells and the plasma levels of HIV RNA at the same time point. **(C)** Correlation analysis between the proportions of NKG2C^+^ NK cells and NKG2A^+^ NK cells, and between the proportions of NKG2A^+^NKG2C^−^ NK cells and NKG2C^+^NKG2A^−^ NK cells. Mann–Whitney tests were used to compare groups. Error bars indicate median and interquartile range. Spearman’s rank test was used to correlate the data, and *p* < 0.05 was considered significant.

Because the proportion of NKG2C and NKG2A expression on NK cells was upregulated in PHI subjects compared with NC subjects, we next investigated whether NKG2C and NKG2A might exert influence on the viral set point, defined as the viral load about 120 days after infection (24, 25). PHI subjects were divided into two groups based on the viral set point: a low set point group (<10^4^ copies/mL) and a high set point group (>10^4^ copies/mL). Our results showed that the proportion of NKG2C^+^NKG2A^−^ NK cells was higher in the low set point group (*p* = 0.0355), while the proportion of NKG2A^+^ NK cells was lower in the low set point group (*p* = 0.0257) (Figure 3A).

We further analyzed the correlation of NKG2C and NKG2A expression with plasma levels of HIV RNA. NKG2C and NGK2A expression and viral load were detected simultaneously in the same samples; results showed that the proportion of NKG2C^+^NKG2A^−^ NK cells was negatively correlated with viral load (*r* = −0.567; *p* = 0.009), and the proportion of NKG2A and NKG2A^+^NKG2C^−^ NK cells was positively correlated with viral load (*r* = 0.650, *p* = 0.002; *r* = 0.457, *p* = 0.043) (Figure 3B). The proportion of NK cells expressing NKG2A was negatively correlated with the proportion of NK cells expressing NKG2C (*r* = −0.662; *p* = 0.002). Moreover, the proportion of NKG2A^+^NKG2C^−^ NK cells was negatively correlated with the proportion of NKG2C^+^NKG2A^−^ NK cells (*r* = −0.805; *p* < 0.0001; Figure 3C).

**Figure 4:**
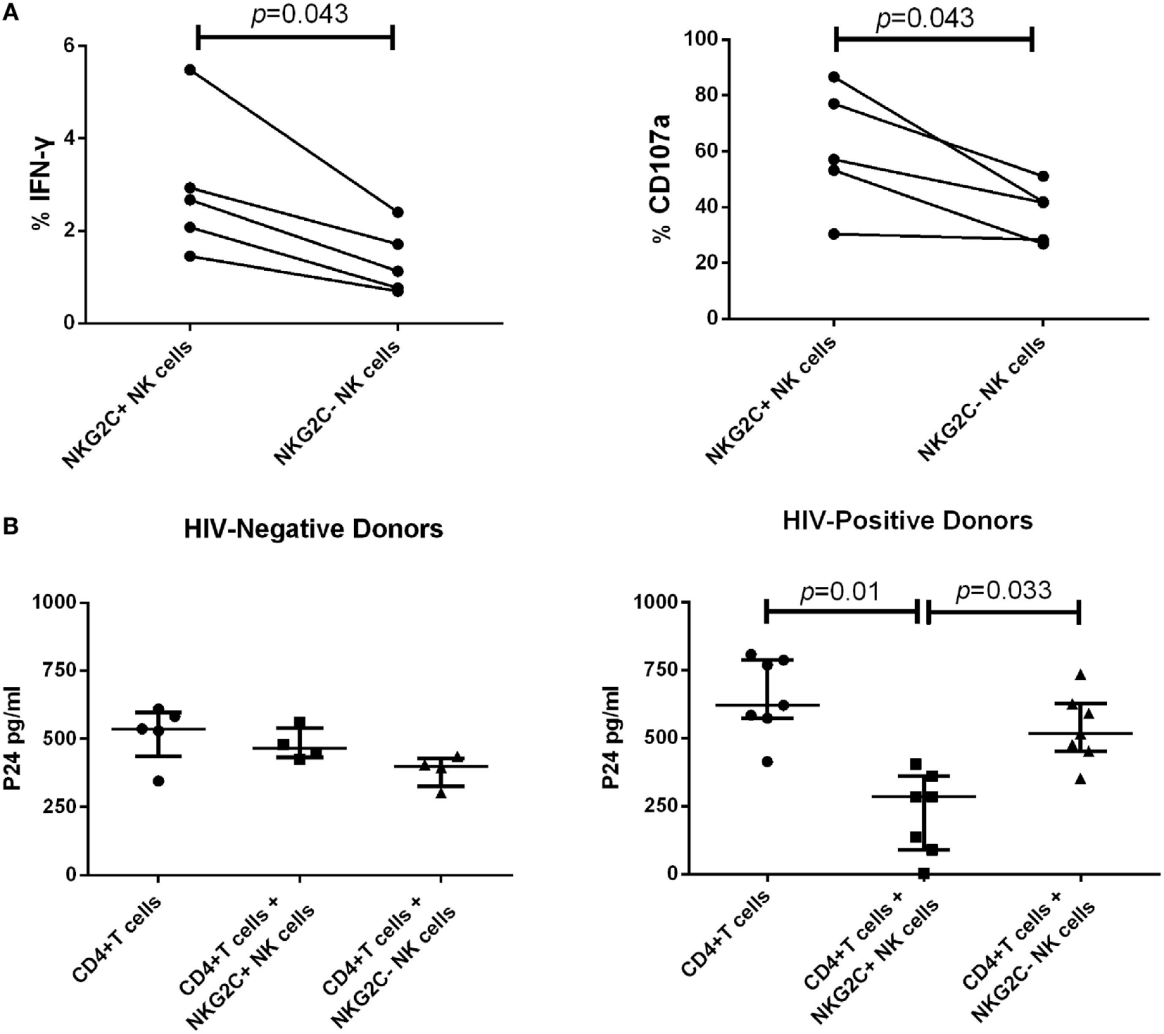
Functional activities of NKG2C^+^ and NKG2C^−^ natural killer (NK) cells. **(A)** Paired comparison of the functional capabilities of NKG2C^+^ and NKG2C^−^ NK cells from HIV-negative donors, in terms of their ability to secrete IFN-γ and express CD107a when stimulated by PMA. **(B)** Comparison of the anti-HIV activity between NKG2C^+^ and NKG2C^−^ NK cells from HIV-negative donors or HIV-positive donors. The P24 antigen was detected in cocultures of HIV-1NL4-3 strain infected CD4^+^ T cells and autologous NKG2C^+^ or NKG2C^−^ NK cells. Wilcoxon matched-pairs signed-rank tests were used to compare the two groups, and the Kruskal–Wallis tests were used to compare the three groups. *p* < 0.05 was considered significant, and error bars indicate median and interquartile range.

Our data showed that during PHI, the elevated proportion of NKG2C^+^NKG2A^−^ NK cells gathered in the group of low HIV set point and was negatively correlated with plasma level of HIV RNA. Therefore, NKG2C could play an important role in anti-HIV activity. We next investigated the secretion of IFN-γ and the expression of CD107a of NKG2C^+^ NK cells in HIV-negative healthy donors. NKG2C^+^ NK cells showed higher IFN-γ secretion and expression of CD107a than NKG2C^–^ NK cells (*p* = 0.043 and *p* = 0.043, respectively; Figure 4A). These results indicated that NKG2C^+^ NK cells possessed strong functional activities. However, whether these highly functional cells could also play roles in the control of HIV infection remains unknown. We further investigated the anti-HIV activity of NKG2C^+^ NK cells. NKG2C^+^ NK cells from HIV-negative donors showed only weak anti-HIV activity (measured by levels of HIV-1 P24 antigen). Remarkably, strong anti-HIV activity was observed in NKG2C^+^ NK cells from HIV-positive donors (*p* = 0.01 and *p* = 0.033, respectively; Figure 4B). Because of the high level of anti-HIV activity exhibited by NKG2C^+^ NK cells, the proportion of NKG2C expression on the surface of NK cells might be used as an important biomarker with which to predict the progression of HIV disease.

### The Proportion of NKG2C^+^NKG2A^−^ NK Cells and the Ratio of NKG2C/NKG2A Predict HIV Disease Progression

**Figure 5:**
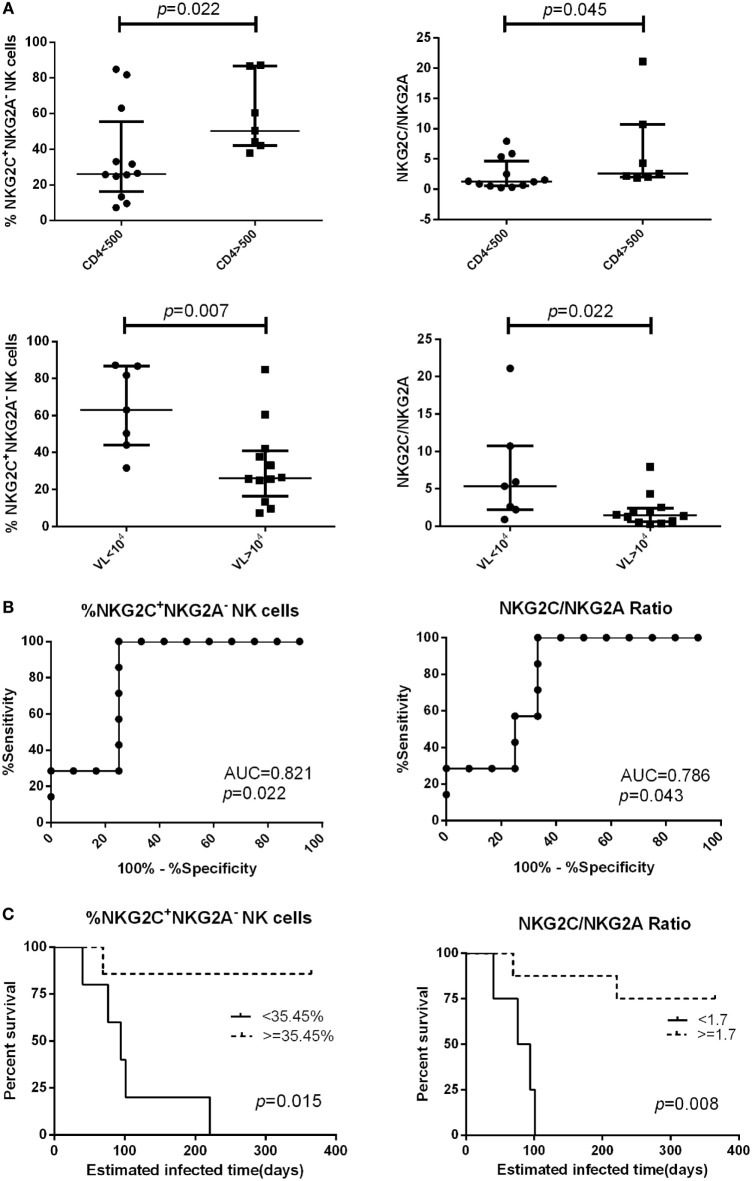
The proportion of NKG2C^+^NKG2A^−^ natural killer (NK) cells, and the ratio of NKG2C/NKG2A, were used to predict HIV disease progression. **(A)** Comparison of the proportion of NKG2C^+^NKG2A^−^ NK cells, or the ratio of NKG2C/NKG2A, at the primary HIV infection (PHI) stage between the CD4 >500 cells/μL group and the CD4 <500 cells/μL group, or between the VL >10^4^ group and the VL <10^4^ copies/mL group at the time of 360-day infection. **(B)** Receiver operating characteristic (ROC) analysis of the proportion of NKG2C^+^NKG2A^−^ NK cells and the ratio of NKG2C/NKG2A. **(C)** Based on the ROC curve for the proportion of NKG2C^+^NKG2A^−^ NK cells, and the ratio of NKG2C/NKG2A, the PHI subjects were divided into a high group (≥35.45% and 1.7, respectively) and a low group (<35.45% and 1.7, respectively). CD4^+^ T cell counts of <500 cells/μL were considered as the end point of follow-up in Kaplan–Meier survival analysis. Mann–Whitney tests were used to compare groups, and *p* < 0.05 was considered significant. Error bars indicate median and interquartile range.

We followed 19 PHI subjects for 720 days and determined their CD4^+^ T cell counts and viral load every 3–6 months. We analyzed the relationship between NKG2A and NKG2C receptor expression on the surface of NK cells from PHI subjects and CD4^+^ T cell counts, or viral loads, at the time of 360-day infection. We found that PHI subjects with a CD4^+^ T cell count >500 cells/μL were associated with a higher proportion of NKG2C^+^NKG2A^−^ NK cells and a higher ratio of NKG2C/NKG2A (*p* = 0.022 and *p* = 0.045, respectively). Furthermore, individuals with a viral load <10^4^ copies/mL were associated with a higher proportion of NKG2C^+^NKG2A^−^ NK cells and a higher ratio of NKG2C/NKG2A (*p* = 0.007 and *p* = 0.022, respectively; Figure 5A). We then analyzed the relative power of the NKG2C^+^NKG2A^−^ NK cell proportion, and the NKG2C/NKG2A ratio, for predicting the progression of HIV disease (CD4^+^ T cell counts), as calculated by ROC analysis. Our results indicated that the NKG2C^+^NKG2A^−^ NK cell proportion had a predictive accuracy of 82.1% for disease progression (CD4^+^ T cell counts), as determined by the area under ROC curve (AUC), while the NKG2C/NKG2A ratio had a predictive power of 78.6% (*p* = 0.022 and *p* = 0.043, respectively; Figure 5B). The point with the largest sum of sensitivity plus specificity was taken as the cutoff point. Based on the ROC curve for the proportion of NKG2C^+^NKG2A^−^ NK cells, and the ratio of NKG2C/NKG2A, cutoff points of 35.45% and 1.7 were selected. Kaplan–Meier survival analysis further showed that the mean time required for CD4^+^ T cell counts to reach <500 cells/μL in the low NKG2C^+^NKG2A^−^ NK cell group (<35.45%) was significantly shorter than the mean time taken for the high NKG2C^+^NKG2A^−^ NK cell group (>35.45%; *p* = 0.015). In addition, the mean time required for CD4^+^ T cell counts to reach <500 cells/μL in the group with a low NKG2C/NKG2A ratio (<1.7) was significantly shorter than in the group with a high NKG2C/NKG2A ratio (>1.7) (*p* = 0.008; Figure 5C).

## Discussion

Natural killer cells are a major component of the innate immune system and are known to exert important antiviral and anticancer effects (12). The activating receptor, NKG2C, and the inhibitory receptor, NKG2A, both belong to the NKG2 receptor family, and bind to the same ligand, HLA-E (26). Viral infections, or tumor generation, may result in alterations in the expression of NK cell receptors and thus affect the function of NK cells (26). HIV infection can also drive changes in the function of NK cells and NK cell receptors. However, few reports have been found about receptors of NKG2C and NKG2A during PHI, particularly with regard to the co-analysis of these two receptors. Our present data indicated that there were some differences between the single-analysis and co-analysis of NKG2C and NKG2A expression on total NK cells and NK subsets. Therefore, it is necessary to consider these two analyses.

In this study, we observed that the proportions of NKG2C^+^ and NKG2C^+^NKG2A^−^ NK cells were significantly higher in PHI subjects than in NC subjects. Previous studies have also reported that cases of HIV chronic infection, co-infected with HCMV, showed an increased proportion of NKG2C^+^ NK cells (22, 27, 28). However, the mechanism for NKC2C elevation after HIV infection is not clear, although an association with synchronous CMV infection has been reported in the literature (29, 30). For example, Brunetta et al. detected HCMV IgG in HIV-infected patients and found that the proportion of NKG2C^+^ NK cells was upregulated in IgG-positive patients (31); however, anti-HCMV IgM and HCMV nucleic acid, indicating current CMV infection, were not tested in previous studies. Our present data showed that PHI subjects without current HCMV infection also had a higher proportion of NKG2C^+^ NK cells, but the anti-HCMV IgG antibody titers in PHI subjects were higher than in the NC group, and the proportion of NKG2C^+^ NK cells was increased in the group with higher anti-HCMV IgG antibody titers. This indicated that the expansion of NKG2C^+^ NK cells was not only associated with HCMV infection.

The viral set point is formed during the acute phase of HIV infection, and our current studies found that the viral set point was related to the proportion of NK cells expressing NKG2C or NKG2A receptors. PHI individuals who had a higher proportion of NKG2C^+^NKG2A^−^ NK cells had lower viral set points, which might be due to increased NK cell functions lack of the inhibition induced by NKG2A receptors. In contrast, PHI individuals who had a higher proportion of NKG2A^+^ NK cells, or NKG2A^+^NKG2C^−^ NK cells, had higher viral set points, indicating that their NK cells had received more inhibitory signals and their function was suppressed.

We also explored the relationship between the proportion of NKG2C^+^ NK cells, or NKG2A^+^ NK cells, and the viral load in PHI subjects. We found that the proportion of NKG2C^+^NKG2A^−^ NK cells was negatively correlated with viral load, while NKG2A^+^ and NKG2A^+^NKG2C^−^ NK cells were positively correlated with viral load. Our results indicated that increased NKG2C expression and decreased NKG2A expression may enhance the ability of NK cells to control viral load in PHI individuals. Thomas et al. reported that during the chronic stage of HIV infection, viral load was positively correlated with NKG2C^+^ NK cells and negatively correlated with NKG2A^+^ NK cells (32). These earlier results differed from the results obtained in our current study, probably due to the very small number of cases in the previous study (NKG2C, *N* = 7; NKG2A, *N* = 5).

In our other experiments, we found that NKG2C^+^ NK cells had a higher proportion of degranulation and IFN-γ secretion when compared with NKG2C^−^ NK cells, indicating that NKG2C^+^ NK cells exhibited greater functional activities. Our investigations of anti-HIV activity demonstrated that NKG2C^+^ NK cells from HIV-positive donors possessed very strong capacities for controlling HIV replication. However, this phenomenon was not apparent in HIV-negative healthy donors. These data suggested that the strong anti-HIV ability of NKG2C^+^ NK cells might be due to NK cell memory. Moreover, HCMV infection might also contribute to the strong anti-HIV ability. Bigley et al. suggested that latent CMV infection could enhance antitumor cytotoxicity *via* the accumulation of NKG2C^+^ NK cells (33). The anti-HIV activity of cases that are co-infected with HIV and HCMV might also be enhanced following the accumulation of high ability NKG2C^+^ NK cells. Therefore, PHI individuals who have a higher proportion of NKG2C^+^ NK cells are more likely to be able to inhibit viral replication and achieve lower viral set points and viral loads.

With a strong capability for anti-HIV replication, NKG2C^+^ NK cells might also influence the prognosis of HIV infection. This study, for the first time, demonstrated that the proportion of NKG2C^+^NKG2A^−^ NK cells, and the ratio of NKG2C/NKG2A, could predict the progression of HIV disease (as evaluated by CD4^+^ T cell counts). However, we found that these parameters could not be used to predict viral load, which remained relatively stable before the AIDS stage, while CD4^+^ T cell counts declined progressively during the prolonged infection period. Therefore, the proportion of NKG2C^+^NKG2A^−^ NK cells, and the NKG2C/NKG2A ratio, may represent significant predictors for the progression of HIV disease.

Overall, during the PHI stage, the population of NKG2C^+^ and NKG2C^+^NKG2A^−^ NK cells expanded and was associated with a low HIV viral set point; furthermore, NKG2C^+^ NK cells exhibited strong cell function and anti-HIV ability. Moreover, the proportion of NKG2C^+^NKG2A^−^ NK cells, and the NKG2C/NKG2A ratio, could act as predictive markers for the HIV disease progression. Our study therefore suggests that NKG2C^+^ NK cells might act as a novel target for HIV immune therapy, and that certain therapeutic strategies might be developed and utilized to increase the proportion of NKG2C^+^ NK cells or to reduce the proportion of NKG2A^+^ NK cells to enhance NK cell function and delay the HIV disease progression.

### Limitations

There was one particular limitation in our study which should be considered when interpreting our results. In our study, the choice of 10^4^ copies/mL, as a limit between low and high viral loads, to compare the proportion or ratio of expression of NKG2A or NKG2C on NK cells, may appear somewhat arbitrary. However, thus far, there are no commonly accepted criteria for viral load grouping, and we therefore selected 10^4^ copies/mL as a limit to group our subjects according to the specific characterization of the patients recruited.

## Ethics Statement

The study was conducted according to the principles enshrined in the Declaration of Helsinki. Each study participant provided written informed consent to take part in the study.

## Author Contributions

All authors listed have made a substantial, direct, and intellectual contribution to the work and approved it for publication.

## Conflict of Interest Statement

The authors declare that this research was conducted in the absence of any commercial or financial relationships that could be construed as a potential conflict of interest.

## Supplementary Material

Figure S1 NKG2C and NKG2A expression on natural killer (NK) cell subsets in primary HIV infection (PHI) and normal control (NC) subjects. Comparison of the proportion of NKG2C^+^, NKG2C^+^NKG2A^−^, NKG2A^+^, and NKG2A^+^NKG2C^−^ on the CD3^−^CD56^dim^CD16^+^ NK subset **(A)**, the CD3^−^CD56^bri^CD16^+/−^ NK subset **(B)**, and the CD3^−^CD56^−^CD16^+^ NK subset **(C)** between NC and PHI subjects.
